# Sex differences matter in the gut: effect on mucosal immune activation and inflammation

**DOI:** 10.1186/2042-6410-4-10

**Published:** 2013-05-07

**Authors:** Sumathi Sankaran-Walters, Monica Macal, Irina Grishina, Lauren Nagy, Larissa Goulart, Kathryn Coolidge, Jay Li, Anne Fenton, Theodore Williams, Mary K Miller, Jason Flamm, Thomas Prindiville, Michael George, Satya Dandekar

**Affiliations:** 1 Department of Medical Microbiology and Immunology, University of CA Davis Health System, 5605A GBSF, 451 Health Sciences Drive, Davis, CA 95616, USA; 2 Student Heath, University of CA, University of California Bldg 588, Santa Barbara, CA, 93106, USA; 3 The Permanente Medical Group Inc, Sacramento, CA, 96185, USA; 4 Department of Gastroenterology and Hepatology, University of CA Davis Health System, 4150 V Street, Suite 3500, Sacramento, CA, 95817, USA

## Abstract

**Background:**

Women and men have diverse responses to many infectious diseases. These differences are amplified following menopause. However, despite extensive information regarding the effects of sex hormones on immune cells, our knowledge is limited regarding the effects of sex and gender on the function of the mucosal immune system. Sex differences also manifest in the prevalence of gut associated inflammatory and autoimmune disorders, including Crohn’s disease, ulcerative colitis and Celiac disease. It is thus hypothesized that a baseline sex-associated difference in immune activation may predispose women to inflammation-associated disease.

**Methods:**

Peripheral blood samples and small intestinal biopsies were obtained from 34 healthy men and women. Immunophenotypic analysis of isolated lymphocytes was performed by flow cytometry. Oligonucleotide analysis was used to study the transcriptional profile in the gut mucosal microenvironment while real-time PCR analysis was utilized to identify differential gene expression in isolated CD4+ T cells. Transcriptional analysis was confirmed by protein expression levels for genes of interest using fluorescent immunohistochemistry. Data was analyzed using the GraphPad software package.

**Results:**

Women had higher levels of immune activation and inflammation-associated gene expression in gut mucosal samples. CD4+ and CD8+ T cells had a significantly higher level of immune activation-associated phenotype in peripheral blood as well as in gut associated lymphoid tissue along with higher levels of proliferating T cells. CD4+ T cells that showed upregulation of IL1β as well as the TH17 pathway-associated genes contributed a large part of the inflammatory profile.

**Conclusion:**

In this study, we demonstrated an upregulation in gene expression related to immune function in the gut microenvironment of women compared to men, in the absence of disease or pathology. Upon closer investigation, CD4+ T cell activation levels were higher in the LPLs in women than in men. Sex differences in the mucosal immune system may predispose women to inflammation-associated diseases that are exacerbated following menopause. Our study highlights the need for more detailed analysis of the effects of sex differences in immune responses at mucosal effector sites.

## Background

Sex and gender-related differences are reflected in the varying magnitude of inflammatory or autoimmune diseases in men and women and are attributed to the diversity of immune responses to infectious or non-infectious stimuli in men and women [1]. Women have a significantly higher incidence of autoimmune diseases than men which are mediated by humoral and adaptive immune responses [1]. It is likely that some of these effects in women are due to the immune-modulatory effects of sex hormones [2-4].

Sex hormones play an important role in the development of adaptive cellular immunity, especially following repeated antigenic stimulus [5]. Gene expression profiling of immune cells from women detected an increased expression of genes associated with inflammatory and cytotoxic T cell responses [5]. However, other studies reported contrasting findings showing increased expression of immune response-related genes in men as compared to women [6]. Discordance among these findings might have been influenced by the timing of the sample collection and the hormonal milieu in women. Sex-specific immune modulation is likely mediated by sex hormones through activation of their specific receptors. Estrogen receptors are expressed on T cells, macrophages and other immune cells that are localized in several tissue compartments in addition to the reproductive tract [7-10]. Estrogen directly modulates immune function through cytokine production, cell activation, and cell proliferation [7-10]. Estrogen is also shown to influence the immune response to tissue injury [11,12]. While an enhanced immune response can be protective against some infections, this could also predispose women to more autoimmune disease [13]. Sex-related differences in antibody production, lymphocyte proliferation, and cytotoxic activity and the influence of steroid hormones on these functions has been previously investigated [13]. Peptide hormones including prolactin, luteinizing hormone (LH), and follicle stimulating hormone (FSH) enhance proliferation of lymphocytes in response to mitogenic stimuli.

Estrogen and progesterone stimulate their cytotoxic activity by enhancing the release of reactive oxygen species [14,15]. Recent gene expression analysis of isolated peripheral blood mononuclear cells (PBMC) from men and women showed a markedly increased expression of immune response related genes in women. Levels of inflammatory and cytotoxic response-associated genes, including IFNγ, IL12Rβ2 and (Leukotriene) LTβ, were all increased in women compared to men [5]. These findings suggest sex-specific differences in T cell function and regulation.

Despite extensive information regarding the effects of sex hormones on immune cells, our knowledge is limited regarding the effects of sex and gender on the function of the gut mucosal immune system [14,15]. Most investigations focused on the analysis of the peripheral blood compartment. However, the gut-associated lymphoid tissue (GALT) is the largest lymphoid organ in the body and is an important site for host-microbe interactions. The involvement of the GI tract is well documented in several inflammatory and autoimmune disorders, including Crohn’s disease, ulcerative colitis, and Celiac disease. It is not known whether the regulation of intestinal inflammatory response is influenced by sex differences [16]. Differences in immune cell infiltration of the colonic mucosa such as mast cells have been shown to correlate with symptomatic differences between the sexes in Irritable Bowel Syndrome (IBS) [17]. However, no differences were observed in healthy controls [17]. The reason for observed gender differences has been attributed to sex hormones and their effects on mast cells, but little is known regarding the effects of sex on lymphocyte function [18]. While many of these studies have been conducted in older women in whom sex hormone levels are low, little is known about sex differences between women and men of younger age groups and if these sex differences play a role in the development of inflammation-associated diseases later on in life.

While studies in the colon have provided evidence for the role of sex in mucosal immune responses, the jejunum is one of the most cellular regions of the small intestine with a large percentage of the body’s T cells. CD4+ T cells are the cellular targets of viral infections such as HIV where gender differences and sex hormones play an important role in pathogenesis and transmission kinetics. Thus, the goal of this study was to investigate the link between sex differences and the regulation of intestinal inflammation by analyzing jejunal biopsies and peripheral blood samples from healthy age-matched male and female participants. Since the gut mucosa is a site of interaction between the gut microbiome and the host immune system, we investigated the effect of sex differences on transcriptional profiles of gut biopsies as well as isolated mucosal CD4+ T cells. Our findings suggest that the sex differences manifest as higher levels of immune activation and inflammation in women compared to men in the absence of any disease or pathology, thus influencing gut mucosal immune response and function.

## Methods

### Sample Collection

Healthy female participants were enrolled in the study at the Center for AIDS Research, Education, and Services clinic and at the Kaiser Permanente Clinic in Sacramento, CA. Male participants were enrolled from the same general geographical area as the female participants. The inclusion criteria included: age between 20 and 60, healthy, and able to participate in an upper GI endoscopy procedure with minimal risk. The exclusion criteria for all participants included: incidence of cardiovascular disease, diabetes, smoking, excessive alcohol consumption, use of illicit drugs or medications. Exclusion criteria specific to female participants was a peri- or post-menopausal state as defined by SWAN [19]. Samples were obtained during the follicular phase of the menstrual cycle (4–7 days following last menstrual period). Menopause is associated with changes in mucosal epithelial function which can further augment observed gender based differences in immune function. Thus postmenopausal state was an exclusion criterion. Age also plays a role in immune responses and to minimize the effects of age all participants enrolled were under the age of 55. Over 40 potential participants were reviewed by the research team and 34 fulfilled the enrollment criteria. The study protocol was approved by the UC Davis Institutional Review Board. All participants provided written, signed informed consent. Twenty milliliters (mL) of blood was collected by venipuncture in EDTA-containing tubes and processed at each time point. Pinch biopsies were obtained by upper GI endoscopy from the jejunum under conscious anesthesia according to previously published protocols [20]. All samples were collected between 7:00 AM and 9:00 AM at the UCD Gastroenterology Clinic, Sacramento, CA after overnight fasting.

### Isolation of peripheral blood mononuclear cells (PBMC)

Whole blood was collected in EDTA-containing tubes and centrifuged for 20 minutes. The white blood cell buffy coat was collected, diluted in 1X PBS, layered over ficoll gradients (Atlanta Biological, Lawrenceville, GA), and centrifuged 20 minutes. The mononuclear cell layer was collected and pelleted by centrifugation. Cells were cultured overnight at 37°C, 5% C0_2_ in culture media (RPMI 1640 supplemented with 10% FBS, 100 U/mL penicillin/streptomycin, and 1% L-glutamine (Gibco) [20]. Five to seven million PBMC were used for CD4+T cell isolation.

### Isolation of intestinal lymphocytes

Jejunum biopsies were placed in isolation media (RPMI 1640 supplemented with 10% fetal bovine serum (FBS), 100 U/mL penicillin, 100 U/mL streptomycin (Gibco, Grand Island, NY) and collagenase type IIb (1 mg/mL) (Sigma, St. Louis, MO) [20] and subjected to shaking at 37°C for four periods of 30 minutes. Cells were washed with RPMI media and rested overnight in culture media (RPMI 1640 supplemented with 15% FBS, 1% gentamycin, 100 U/mL penicillin, 100 U/mL streptomycin, 1% L-glutamine, 1% sodium pyruvate, 2.5% Hepes, and 0.5% amphotericin B (Gibco)) at 37°C with 5% CO_2_. Cells were filtered through a 40 μm filter the next day and counted. Three to four million lymphocytes were filtered the same day for further CD4+T cell isolation.

### CD4+ T cell isolation

Isolated lymphocytes from the jejunum and peripheral blood were enriched for CD4+T cells by magnetic bead sort (Dynal, Carlsbad, CA). Magnetic beads pre-coated with CD8 antibody were incubated with isolated lymphocytes for 30 minutes at 4°C on a gentle rotating platform and CD8 lymphocytes were collected by magnet and incubated with CD13 coated magnetic beads. Pan-mouse IgG beads (Dynal) were first incubated for 30 minutes at 4°C on a rotating platform with monoclonal IgG CD13 antibody (BD, San Jose, CA), and then CD13 coated beads were incubated with CD8 depleted lymphocytes for 30 minutes at 4°C on a gentle rotating platform. The CD8^-^ CD13^-^ lymphocytes were collected by magnet, washed in culture media, and incubated overnight at 37°C/5%CO_2_ at a maximum concentration of 2 million cells/ml. Cells were pelleted the following day and stored in -80°C until use. Supernatant from overnight culture was also stored at -80°C until needed. Purity of PBMC (90-100%) and jejunal lymphocytes (65-85%) was determined by flow cytometry.

### Immunophenotypic analysis

Isolated lymphocytes (1 million cells/tube) were incubated with Aqua (Amcyan) Live/Dead Dye (Invitrogen, Carlsbad, CA) (0.5 uL/mL 1XPBS + 1million cells). Lymphocytes were then washed with staining buffer (filter sterilized 1XPBS with 3% heat-inactivated FBS and 0.1% sodium azide). Cells were incubated with 10% normal mouse serum (Jackson Immunoresearch Laboratories, Inc) and then with fluorescent mouse anti-human monoclonal antibodies. CD3 (Beckton Dickinson, Mountain View, CA), CD45RA, CD45RO, CD8 (Invitrogen), CD95 (Biolegend, San Diego, CA), CD28, CD4, HLADR, Ki67 (E-Bioscience) antibodies were used. Cells were washed and fixed with 1% paraformaldehyde (PFA) (Sigma) prior to analysis on the LSR II flow cytometer (Beckton Dickinson at the UC Davis core facility). The cells were first gated based on negative staining with live dye, followed by a lymphocyte gate. These populations were further analyzed by specific staining. A minimum of 500,000 events was collected per sample and data was analyzed using Flow Jo (Tree Star, Inc. San Carlos, CA).

### Stimulation assays

Isolated lymphocytes (~1 million cells) were stimulated with phorbol 12-myristate 13-acetate (PMA) (1 ng/mL) + Ionomycin (1 μM/mL) (Sigma), or negative control media alone at 37°C with 5% CO_2_ for 6 hours. Anti-CD28 pure monoclonal antibody (Ebioscience) was added to aid co-stimulation, followed by addition of Brefeldin A (Sigma) for the final 5 hours to prevent Golgi transport of cytokines out of cells. Cells were washed with 1X PBS/1%BSA, stained for surface markers, and fixed in 1% PFA. Fixative was washed out and cells were permeabilized with Caltag Permeabilization Solution B (Invitrogen) and stained with monoclonal antibodies IFNγ, IL-2, and TNF-α (BD, Ebioscience) for 20 minutes at room temperature. Samples were washed and fixed with 1% PFA before analysis with LSR II. A minimum of 500,000 events were collected for each sample and analyzed using Flow Jo software. Polyfunctional cell analysis was performed using SPICE (v4.1.6) and PESTLE (v1.5.4) software courtesy of Mario Roederer, Vaccine Research Center, NIAID/NIH, Bethesda, MD.

### Transcriptional analysis

Gene expression profiles in gut biopsies from participants were analyzed using microarray technology and real-time PCR. In brief, total RNA was isolated from cryopreserved jejunal tissue utilizing protocols and reagents in the RNeasy RNA isolation kit (Qiagen). Messenger RNA amplification, labeling, hybridization to human whole-genome U95av2 GeneChips© (Affymetrix) staining and scanning were performed as previously described [21,22] utilizing kits and protocols described in the Affymetrix Gene Expression Analysis Technical Manual. Microarray data were analyzed cross-sectionally amongst all groups utilizing the dChip (http://www.hsph.harvard.edu/cli/complab/dchip/) software program. A minimum fold-change of 1.5 (p-value ≤ 0.05) between groups was established as a cut-off criterion for statistical validation. Samples from male participants were used as baseline values for identifying differentially expressed genes (DEG) in samples from female participants. Pathway analysis was performed using Ingenuity Pathway Analysis (IPA) (http://www.ingenuity.com) software.

RNA was also isolated from CD4+ T cells enriched from PBMC as well as LPL using the same methods as in the microarray analysis. CDNA was synthesized using Superscript III (Invitrogen, CA) and used in Taqman© Real-Time PCR assay as previously described (Applied Biosystems, CA) [21,22]. The data were normalized to the housekeeping gene GAPDH and calibrated to the average of the male samples from the same tissue source. The data were presented as a fold increase in RNA level compared to the calibrator [23].

### Immunohistochemistry

Immunohistochemical analysis (IHC) was performed to detect and localize expression of IL17, TNFα, FoxP3 and IL1β in the small intestinal mucosa. Jejunal biopsies were embedded in paraffin blocks following fixation with 4% PFA. Tissue sections (5 μm) were incubated with polyclonal anti-IL17, anti-TNFα, anti-FoxP3 or anti-IL1β overnight at 4°C, followed by incubation with FITC-labeled secondary antibodies for 1 hour as previously described. Sections were mounted using SlowFade with Dapi (Invitrogen, Carlsbad, CA). Images were captured by confocal laser microscopy using LSM 5 and PASCAL software (Zeiss, New York) and then analyzed using Image J software, NIH, Maryland.

### Statistical analysis

Data sets were analyzed using the two-tailed paired or unpaired *t*-test as applicable, taking into account the distribution of the data (GraphPad Prism version 5.00 for Windows, GraphPad Software, San Diego California USA, http://www.graphpad.com). Due to the limited availability of samples to perform all the analyses, a selected number of samples were utilized in each assay over 75% overlap between most experiments. The real-time data were analyzed for statistically significant differences at the level of the calibrated values as well as at the level of the linearized fold change representing relative expression.

## Results

### Demographics of study participants

Thirty-four study healthy participants were enrolled over a period of 5 years; 22 were female, and 12 were male (Table 1). Women were 23 to 52 years old, had not had chemotherapy or radiotherapy, had intact ovaries and uterus, and were not on hormonal contraceptives during or prior to sample collection. Men were age-matched to female participants. There were no significant differences between plasma estradiol levels within the group of women. Female participants had a mean plasma estradiol level of 126 pg/ml (Range 102-140 pg/ml). Men and women did not differ significantly in age or race/ethnicity distributions. All participants completed a health survey prior to sampling. No significant differences were observed in the answers and all participants appeared to be healthy; however latent viral infections could not be ruled out.

**Table 1:** Characteristics of participants

**Group ID**	**Male**	**Female**
**Number**	**12**	**22**
**Age Range (yrs)**	**21**-**57**	**23**-**52**
**Race/Ethnicity**		
**White**	**6**	**15**
**Hispanic**	**1**	**1**
**Black**	**3**	**6**
**Asian**	**2**	**0**

### Baseline gut mucosal gene expression shows increased immune activation and inflammation in women compared to men

**Figure 1:**
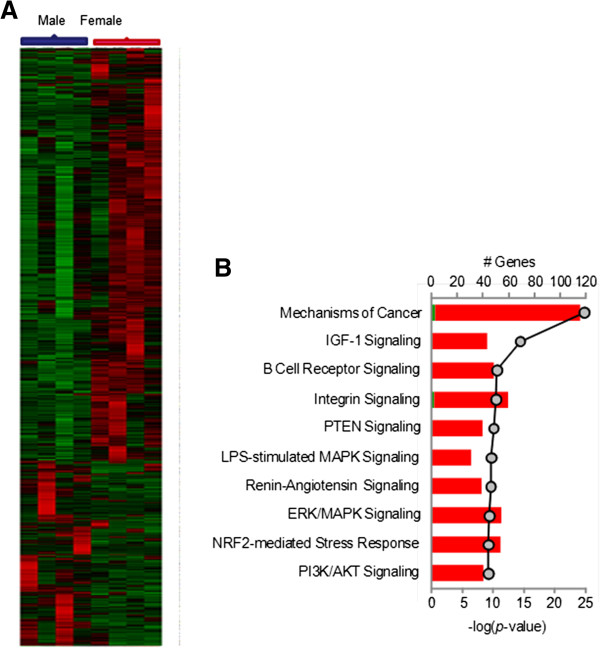
**The microenvironment of the gut in women has a transcriptional profile indicative of increased activation and turnover. **Transcriptional analysis of jejunal mucosa was performed using oligonucleotide microarray analysis. (**A**) All samples cluster in accordance with the gender of the participant. A large portion of the gene expression included genes that were upregulated in women as compared to men (**B**) The genes that were upregulated clustered into functional categories of IGF-1 signaling, Integrin Signaling and other signaling pathways (p<.05).

The transcriptional profile of the gut microenvironment was studied by oligonucleotide microarray analysis. Hierarchical cluster analysis showed that the samples from male participants were similar in their gut mucosal gene expression profile and clustered together, while the samples from female participants clustered together (Figure 1A). In general, the majority of gut mucosal genes that were differentially expressed between men and women were expressed at higher levels in women as compared to men (1903 increased in women vs 889 increased in men). Further analysis of the 1903 upregulated genes in women grouped them in statistically significant functional clusters that included genes associated with cancer, IGF-1 signaling as well as other immune signaling pathways (Figure 1B) (p<0.05). Between 20 and 100 different gene transcripts were identified within these pathways, indicating a pattern of gene regulation associated with activation in the gut microenvironment.

**Figure 2:**
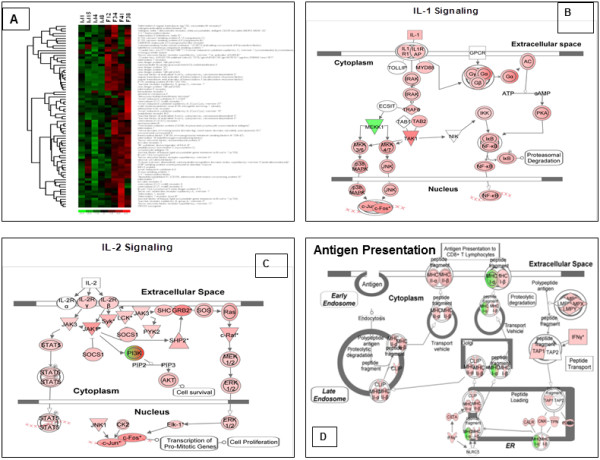
**Pathways associated immune activation are upregulated in the gut mucosa in women.** (**A**) A large cluster of the increased levels of gene transcription in the mucosal microenvironment includes genes and pathways associated with immune activation, antigen presentation and inflammation (M: male, F:female). Specific pathways that were upregulated in the microenvironment of the female gut mucosal tissue over baseline of male mucosal tissue include (**B**) IL1 Signaling, (**C**) IL2 Signaling and (**D**) Antigen presentation (Red: Increase in expression, Green: decrease in expression).

**Figure 3:**
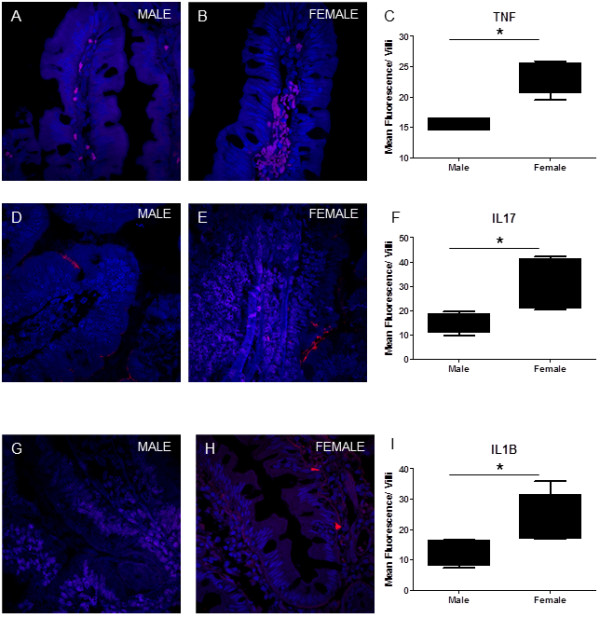
**Immunohistochemical analysis of jejunal mucosal samples. **Tissue sections were stained for TNFα, IL17 and IL1β. A higher number of cells were observed that were positive for TNFα in women (**3B**) compared to men (**3A**). Quatitation for the mean fluorescence intensity of the tissue section showed similar results (**3C**). Women also had higher levels of IL17 + cells in the villus compared to men (**3E **vs **3D**). On quantitation of mean fluorescence intensity within the villus, women had significantly higher levels compared to men (**3F**). IL1β staining in the jejunal mucosa in women was higher than in men (**3G**, **H **and **I**). No staining was observed in antibody negative controls. All images were obtained at a 63X magnification. (* = p<.05). Nucleus in blue; Antibody Positive cell in red.

The largest cluster of genes that was increased in abundance in the microenvironment of the gut in females included genes involved in inflammation and immune activation (Figure 2A). Further analysis of these genes showed that the IL1 signaling cascade was upregulated with an increase in expression of IL1, IL1R, MY88 and NF-κB (Figure 2B). Increased levels of IL 1β were also observed at the protein level by immunohistochemical analysis in women compared to men (Figure 3G, H, I). IL2 receptor β and γ expression was increased in women compared to men. Genes belonging to the STAT3 signaling pathway as well as SOCS1 were also upregulated in women (Figure 2C). Interestingly, multiple gene transcripts involved in antigen presentation were also expressed at higher levels in women as compared to men. Further detailed analysis of the transcriptional data indicated that the downstream effectors of CD8+ T cell function, such as IFNγ were also increased at the transcriptional level in women. Many of these changes may be attributed to small differences in the cellular population; however no significant differences were observed in actual percentages of lymphocytes between men and women.

**Figure 4:**
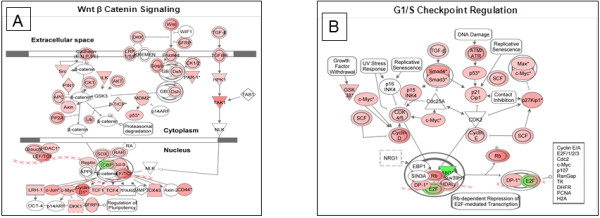
**Microarray analysis also indicated an increase in Wnt signaling and cell cycle regulation in women. **(**A**) Increased turnover of epithelial cells is associated with an increase in expression of genes in the Wnt β Catenin signaling pathway with downstream nuclear effectors such as SOX, HDAC1. (**B**) A compensatory increase in cell cycle regulation via the G1/S Checkpoint was also observed at the transcriptional level. (Red: Increase in expression, Green: decrease in expression).

Increased levels of RNA transcripts associated with cellular proliferation were observed in women compared to men. These transcripts could be reflective of the proliferative/regenerative nature of the gut microenvironment. Wnt β-Catenin signaling pathway is important in epithelial regeneration and this pathway was transcriptionally up regulated in the jejunal mucosa in women (Figure 4A). Also involved in cell cycle regulation, the G1/S checkpoint regulation associated genes were increased in women (Figure 4B). Thus, it appeared that increased transcription in the gut mucosa in women was association with cell proliferation and immune activation.

### Women have higher levels of T cell proliferation and T cell activation in peripheral blood and gut of women in the absence of disease

**Figure 5:**
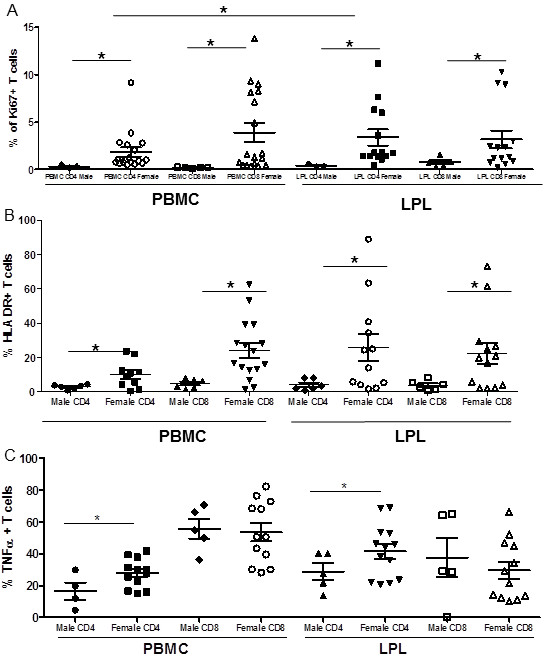
**Women have higher levels of Ki67+ T cells as well as activated T cells in peripheral blood and GALT as measured by flow cytometry. **(**A**) Ki67+ cells are in the process of proliferation. (**B**) Higher levels of T cell activation in women as compared with men in peripheral blood and GALT measured as % of HLA-DR+ T cells. (**C**) Following PMA ionomycin stimulation a higher percentage of CD4+ T cells produce TNFα in women than in men. (* = p<.05).

To identify the cells contributing to increased immune activation and the proliferation profile observed in women, T cell proliferation in the peripheral blood and gut mucosa was measured by flow cytometry using Ki67 antibody. Higher levels of Ki67+ CD4+ T cells were observed in women compared to men in peripheral blood (PBMC 1.852% vs .3133%) (Figure 5A). All values shown are the means of the values in each group. Interestingly, within women the level of CD4+ Ki67+ T cells was significantly higher in Lamina Propria Lymphocytes (LPLs) than in PBMCs which may be indicative of the increase in activation state in the LPLs as compared to PBMCs (mean 3.386% vs 1.852%) (p=.011). Women also had higher levels of proliferating CD4+ T cells the gut (LPL: 3.386% vs .42%) compared to men. Women, also had significantly higher levels of Ki67+ CD8+ T cells in the LPLs as well as in PBMCs compared to men (PBMC: 1.775% vs .14%, LPL: 3.181% vs .77%). A positive correlation was observed between CD8+ HLADR and Ki67 expression in male LPL but not in PBMC or in samples from women. The data in women appeared to have a greater spread than in men. No significant differences in general health were observed within the group of females participants.

The percentage of T cells expressing the activation marker, HLA-DR, was determined using flow cytometry (Figure 5B). The percentage of activated CD4+ T cells in peripheral blood of women was significantly higher than in men (p=.0014). The percentage of HLA-DR expressing activated CD8+ T cells was also increased in women compared to men with a mean difference of 19.23 (p=.0006). This increase in T cell activation in peripheral blood is also reflected in GALT. The percentage of cells that were CD4+ HLA-DR+ in the LPL was also higher in women compared to men, indicating that cell activation in women was higher than in men (p=.0195), and CD8+ T cell activation was also higher in women compared to men (p=.0078). A positive correlation (p>.05) was observed between CD4+ HLADR and CD8+ HLADR expression in all groups in PBMC as well as LPL.

LPL derived CD4+ T cells from women are also more sensitive to non-specific stimulation with PMS/Ionomycin as compared to men. LPL and PBMC were stimulated with PMA/Ionomycin and TNFα levels were measured by intracellular staining and flow cytometry (Figure 5C). In general, a higher level of responsiveness was observed in LPLs of all study participants, male and female. However, CD4+ cells from women had higher levels of TNFα in the LPLs than in men (41.61% vs 28.8%) (p<0.05). No significant differences were observed in CD8+ T cells from the blood or gut. Surprisingly, the percentages of CD8+ T cell producing TNFα on stimulation in the gut was significantly lower than that in the peripheral blood in women, but not in men. Increased expression of TNFα protein was also detected the gut tissue of women by immunohistochemistry (Figure 3A, B and C).

### Gut mucosal CD4+ T cells contribute to the elevated Th17 associated inflammatory profile in women

**Figure 6:**
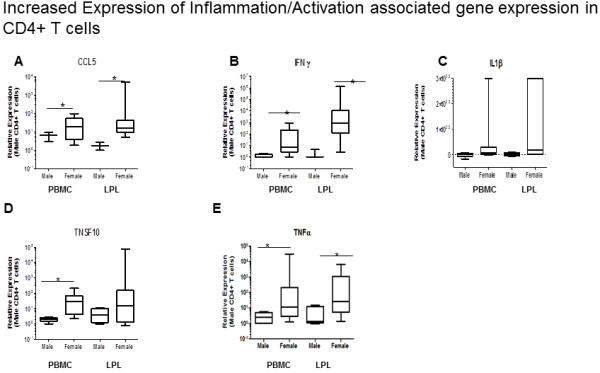
**The activation profile seen in peripheral blood and gut is matched by the increase in transcription of activation and inflammation associated genes (CCL5/RANTES, IFNγ, TNSF10 and TNFα, IL1β) in CD4+ T cells of women compared to men. **Real Time PCR analysis of CD4+ T cells enriched from the PBMC and LPL of women showed a significant increase in transcription of proinflammatory factors such as CCL5 (**A**), IFNγ (**B**), IL1β (**C**), TNSF10 (**D**), and TNFα (**E**). (* = p<.05).

**Figure 7:**
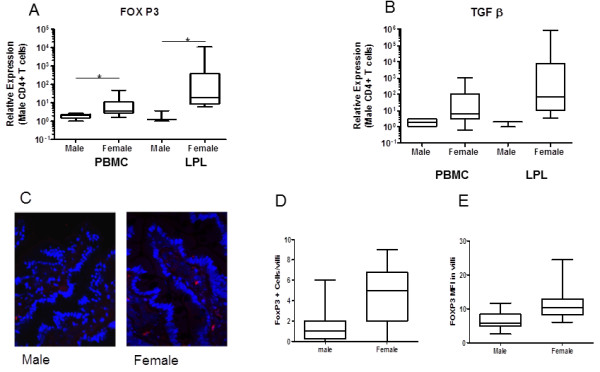
**Increased expression of Treg associated genes in enriched CD4**+ **T cells in females compared to males. **The regulatory effects of CD4+ T cells are necessary to balance the inflammation and immune activation normally stimulated by CD4+ T cells. . FOXP3 and TGFβ expression are hallmarks of Regulatory T cells. Transcriptional analysis by Real-Time PCR indicates that (**A**) FOXP3 is expressed at higher levels in women compared to men as well as TGFβ (**B**). (* = p<.05). Increased number of FOX P3 positive cells were also observed by IHC with higher MFI in women compared to men (C, D, E) (Nucleus in blue, FOX P3 in Red).

CD8 T cells from women, despite having higher levels of HLA-DR compared to men, showed lowered responsiveness to non-specific stimulation. CD4+ T helper cells play an important role in the adaptive immune system, especially in the gut. To determine if the CD4+ T cells contributed to the immune activation in women, mRNA profiling was performed in CD4+ T cells enriched from peripheral blood and the gut mucosa. Increased transcript levels of CCL5 (RANTES) were detected in isolated CD4+ T cells from the blood and gut (Figure 6). In addition to CCL5, IFNγ and TNFα transcript levels (Figures 7) were also significantly increased in women compared to men as measured by real-time PCR assay, as well as at the protein level as detected by flow cytometry and immunohistochemistry (Figure 3C, 6A, B and C) Tumor necrosis factor ligand superfamily 10 is a factor associated with cell death. The binding of this protein to its receptors has been shown to activate of MAPK8/JNK, caspase 8, and caspase 3, thus inducing cell death. TNFS10 transcript levels increased in the blood but not significantly in the gut. IL1β transcripts were increased in the microenvironment of the gut mucosa as well as in isolated CD4+ T cells. It is likely that the activated CD4+ T cells contribute to the inflammatory profile observed in women in the gut as well as in peripheral blood.

The CD4+ T cells also showed a significant increase in expression of FOXP3 as well as TGFβ in women compared to men (p<.05) (Figure 7). In both cases the LPLs tended to have higher expression than PBMC-derived CD4+ T cells, but this increase was not statistically significant. These observations could be due to a compensatory response to increased immune activation in CD4+ cells. IHC for Fox P3 demonstrated significantly higher numbers of FOXP3+ cells in villi of women compared to men as well as increased expression in positive cells (Figure 7C, D, E).

**Figure 8:**
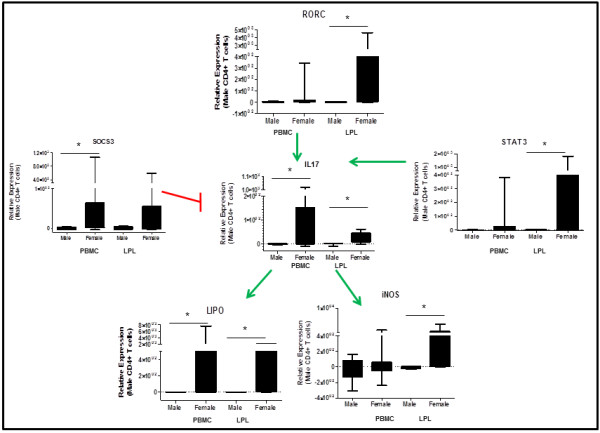
**Upregulation of IL17 pathway associated genes in women compared to men in peripheral blood and gut CD4+ T cells. **This transcriptional profile in CD4+ T cells in the blood and gut may contribute to inflammation and immune activation in the gut in women. (* =p<.05).

The gut mucosa is a site of complex host-microbe interactions. The Th17 response is a characteristic of an effective immune response against pathogenic bacteria present in the intestinal lumen. IL17 is induced by a complex signaling pathway and contributes to inflammation. IL17 expression is several-fold higher in CD4+ T cells from women as compared to men (128 fold in CD4+ T cells in PBMC, 26-fold in CD4+ T cells in LPL). RORγ (RORC) and STAT3 are upstream inducers of IL17, while Lipocalin and iNOS are downstream inducers of inflammation whose expression depends on IL17. These genes are expressed at higher levels in women compared to men, especially in CD4+ T cells derived from the gut. SOCS3, a negative regulator of IL17 is also induced, likely in response to the strong induction of IL17 in women (Figure 8). IL17 levels in gut tissue were also increased in women, which were localized to the lamina propria and to a lesser extent the epithelium (Figure 3D, E and F). However, there was no significant correlation between the expressions of Th17 associated genes.

## Discussion

Sexual dimorphism of the immune system plays an important role in disease occurrence, host-microbe interactions, and mechanisms of pathogenesis [6]. Females respond to infections with a more robust innate and adaptive immune response compared to males. This, however, is a double-edged sword. For some infections, this can be beneficial because it results in faster clearing of the pathogen. In others, such as H1N1 influenza virus, the robust immune response may result in a greater number of fatalities in females compared to males. Increased immune activation may potentially place females at a greater risk of developing autoimmune disorders.

In this study, we demonstrated an upregulation of gene expression related to immune function in the gut microenvironment of women compared to men in the absence of any current disease or pathology. Upon closer investigation, CD4+ T cell activation levels were higher in the LPLs in women than in men. Prior studies have demonstrated an increased immune response in women following nonspecific stimulation of the peripheral blood [5]. However, the gut mucosal immune system is the largest in the body. The gut is also the most important site of host-microbe interactions. It is likely that the heightened responsiveness in the gut is triggered by the need to reduce the systemic load of microbial translocation and may be driven by gender associated differences in that process. Our study demonstrated sex differences in the mucosal immune system in which women had a higher baseline level of immune activation compared to their male counterparts thus predisposing them to inflammation-associated diseases that are exacerbated following menopause.

Studies in murine models of burn injury indicate that higher estrogen levels in female mice contribute to improper cell-mediated immune response to injury with an aberrant inflammatory response that increases mortality. In contrast, male mice are immunosuppressed following hemorrhagic injury and are more likely to suffer from sepsis than female mice with a comparatively enhanced cellular immune response under the same conditions [24]. In our study, healthy women had an enhanced inflammatory response (IL1β pathway) as well as an immune proliferative response (IL12 pathway) in the small intestinal mucosa, which may be protective in some cases of injury. Furthermore, an increase in TNFα and IFNγ, likely to originate from the T cells in the LPLs following mitogenic stimulation, was evidence of a robust cellular immune response in women as compared to men in our samples. This occurred both at the level of the gut microenvironment as well as the cellular level in CD4+ T cells.

Prior studies reported that following mitogenic stimulation, IL17A expression was upregulated in men compared to women in PBMC [5]. In our study, we showed a steady state increase of IL17A expression in CD4+ T cells of women compared to men. IL17A is produced by both CD4+ and CD8+ T cells. In a limited number of patient samples, increased IL17 MFI following mitogenic (PMA/Ionomycin) stimulations was detected in PBMC of men than of women (data not shown). In CD4+ T cells, expression was increased of transcription factors upstream of IL17A acivation, such as RORC and STAT3, as well as downstream effectors such as iNOS and Lipocalin. Th17 responses in infectious diseases, such as HIV infection, are important in controlling mucosal pathogenesis. In animal models, basal Th17 levels may play a role in disease progression. A basal gender-based difference in TH17 expression may contribute to gender differences observed in disease progression in viral and bacterial infection. This increase in baseline cellular immune response could be protective during infections but the lack of resolution may be detrimental in the development of autoimmune diseases.

Estradiol in women plays an important role in the repair and regeneration of mucosal surfaces. In healthy women, the Wnt signaling pathway is upregulated, indicating an increase in turnover and regeneration of the gut mucosa, likely of the epithelial cells. Wnt/β Catenin signaling is important for the differentiation and maturation of gut epithelial stem cells into mature enterocytes as well as Paneth cells in crypts. The increase in expression of transcripts belonging to this pathway could also support the concept of increased proliferation and turnover of epithelial cells in women as compared to men. This may also indicate baseline differences in epithelial integrity between males and females. These findings warrant further investigation.

Despite the small sample size in our study, it has identified inherent differences in the gut mucosal immune system based on the sex and gender of the participant and has pointed to an important concept relevant to large-scale patient studies of drug efficacy or immune modulators. It was not feasible to perform all cellular and molecular analyses on all samples due to limitations associated with sample collection. It was beyond the scope of this study to identify latent viral infections such as EBV and CMV that may drive immune activation in health adults and can be investigated in future studies. The gut microbiome also plays an important role in the immune homeostasis. While microbiome differences may exist between the sexes, such studies have not been performed in detail. Thus, similar studies using animal models may be necessary to further delineate the effects of sex on gut mucosal function. Our study is the first study to demonstrate the baseline differences in the function of GALT, the largest lymphoid tissue in the body.

Taken together with the knowledge that sex-based differences in the immune system play an important role in disease progression in infectious disease and vaccine responses, sex of the study population should be considered during clinical trials [25]. Our study highlights the need for more extensive and detailed analysis of the effects of sex differences in immune responses at mucosal effector sites.

## Competing interest

The authors have no conflict of interest or financial or non-financial competing interests to disclose.

## Authors’ contribution

SSW is the PI on the study, planned and carried out the study and manuscript preparation. MM performed and analyzed immunophenotypic analysis. IG performed and analyzed oligonucleotide microarray analysis. LN performed and analyzed immunophenotypic analysis. LG performed sample preparation and real-time PCR. KC performed sample preparation and real-time PCR. JL performed and analyzed IHC. AF performed and analyzed IHC. TW performed and analyzed IHC. MM collected samples and helped with manuscript preparation. JF collected samples and helped with manuscript preparation. TP is the attending gastroenterologist and performed the EGD’s. MG is the expert for analysis of microarray data. SD is the co-PI on the study. All authors read and approved the final manuscript.
